# Prognostic value of ICU-acquired hypernatremia in patients with neurological dysfunction

**DOI:** 10.1097/MD.0000000000003840

**Published:** 2016-09-02

**Authors:** Bei Hu, Qianpeng Han, Nashun Mengke, Kairan He, Yiqin Zhang, Zhiqiang Nie, Hongke Zeng

**Affiliations:** a Southern Medical University, Guangzhou; b Department of Emergency and Critical Care Medicine, Guangdong General Hospital, Guangdong Academy of Medical Sciences, Guangzhou; c Department of Cardiovascular Epidemiology, Cardiac Surgery, Guangdong Cardiovascular Institute, Guangdong General Hospital, Guangdong Academy of Medical Sciences, Guangzhou, Guangdong, China.

**Keywords:** critically neurological patients, ICU-acquired hypernatremia, prognosis

## Abstract

Many studies have indicated that hypernatremia is associated with increased mortality. In this study, we aimed to explore the relationship between intensive care unit (ICU)-acquired hypernatremia and the prognosis of critically neurological patients.

Based on serum sodium level in the ICU, 450 patients were divided into 3 groups: 222 had normal serum sodium, 142 had mild hypernatremia, and 86 had severe hypernatremia. Kaplan–Meier and multivariable binary logistic regression analyses were performed to evaluate the prognostic value of hypernatremia in critically neurological patients. Receiver operating characteristic (ROC) curve was constructed for serum sodium levels to determine their roles in predicting ICU mortality.

Hypernatremia was significantly related with age, Glasgow Coma Scale (GCS) score, serum sodium, APACHE II score, and serum creatinine. Moreover, the different treatment outcome including mechanical ventilation, the days of stayed in ICU, and Glasgow Outcome Scale score had correlation with serum sodium levels. Old ages, GCS score, therapeutic intervention scoring system (TISS) score, APACHE II score, serum sodium peak, and so on were all associated with the mortality. In addition, hypernatremia was an independent prognostic factor for critically neurological patients by logistic regression analysis (odds ratio = 1.192, 95% confidence interval = 1.135–1.252, *P* = 0.000). Moreover, we got the sensitivity of 79.4% and specificity of 74.5% in the ROC analysis between peak serum sodium and the mortality. The area under the ROC curve was 0.844, and the optimal cutoff value was 147.55.

Our results showed that ICU-acquired hypernatremia may be a potential prognosis marker for critically neurological patients.

## 1 Introduction

Hypernatremia is an electrolyte imbalance condition at various stages in the occurrence and development of many diseases, and usually it can be diagnosed as hypernatremia when the serum sodium concentration is more than 145 mmol/L.^[1,2]^ Hypernatremia is also the ubiquitous severe electrolyte imbalance in the intensive care unit (ICU). Studies showed that about 2% to 9% patients in ICU have hypernatremia.^[3]^ In addition, hypernatremia is more frequently found in the neurosurgical ICU than in the general ICU.^[4]^ The incidence and mortality of hypernatremia are both high in severe traumatic brain injury (TBI). Also in the patients with cerebral hemorrhage and cerebral infarction, the incidence of hypernatremia is 6% and 3%, respectively.^[5]^ In general cases, according to the occurred time, hypernatremia can be divided into hypernatremia present at admission to the ICU and hospital-acquired hypernatremia. Polderman et al^[6]^ found that hospital-acquired hypernatremia patients had a higher mortality rates than those with hypernatremia present at admission to the ICU (32% and 20.3%, respectively).

Many studies found that levels of serum sodium and speed of serum sodium elevated directly determined the severity of hypernatremia patients and their prognosis. Once the hypernatremia has not been timely controlled, it may cause cognitive impairment and epilepsy, even lead to death. At the early stage of hypernatremia, there are no obvious clinical symptoms, and clinicians are not easy to find only by clinical manifestation. Hypernatremia acquired in the ICU has been regarded as an indicator of the quality of ICU care, because its origin is often iatrogenic.^[6]^ It was reported that hypernatremia acquired in the ICU was an independent risk factor for mortality in critically ill patients.^[7,8]^ So early detection and timely treatment of hypernatremia are related to the prognosis of critically ill patients, and the prevention of hypernatremia also becomes the focus of scholars at home and abroad.

In our study, we aimed to determine the incidence and clinical feature of critically neurological patients who were with ICU-acquired hypernatremia, discuss the influencing factors of ICU-acquired hypernatremia, and analysis the effect of ICU-acquired hypernatremia to the prognosis of critically neurological patients.

## 2 Materials and methods

### 2.1 Subjects

From January 1, 2011 to December 31, 2013, a total of 2426 critically neurological patients were retrospected from the ICU 1 area and 2 area of Guangdong General Hospital. Our study was approved by the Ethics Committee of Guangdong General Hospital, Guangdong Academe of Medical Sciences and obtained the exemption informed consent. At last, 450 patients conformed to the standards were enrolled in this study.

In this study, the inclusion criteria were as follows: more than 18 years old; admitted to neurologic ICU (NICU) for severe nervous diseases, and stay of more than 24 h; and normal serum sodium levels when admission in the NICU. And the exclusion criteria were as follows: renal insufficiency; patients with pituitary adenoma or craniopharyngioma; severe infection, major organ injuries, hemodynamic unstable, and hypovolumic shock; frequent diarrhea; hyponatremia after in NICU; and incomplete information.

### 2.2 Measurements

The following basic information of patients was recorded, including age, gender, history of hypertension, time and place of NICU admission, diagnosis, vital signs as well as laboratory data, including Glasgow Coma Scale (GCS) score, APACHE II score, and therapeutic intervention scoring system (TISS) score. Moreover, therapy-related information including intake and output record, drugs, surgery, mechanical ventilation, nutritional support, and so on were also collected. In ICU, the laboratory data, such as electrolytes, renal function, blood glucose, and serum sodium, were recorded clearly. About the prognosis data, length of ICU and hospital stay, cost, length of mechanical ventilation, Glasgow Outcome Scale (GOS) score of leave ICU and hospital, and mortality during ICU and hospital were all recorded for this study.

### 2.3 Statistical analysis

All statistical analyses were performed using the software of SPSS 19.0 and GraphPad Prism 5. The data are summarized and presented as means ± standard deviation. *T* test was used for the comparison between the measurement data. The counting data were detected by Chi-squared test. Kaplan–Meier curve was performed to assess the survival of critically neurological patients with varies degree of hypernatremia when died. Multivariable binary logistic regression analysis was used to determine the influence of hypernatremia on mortality. To determine the serum sodium-level distinguished nonsurviving from surviving patients, receiver operating characteristic (ROC) curves were constructed for serum sodium levels on admission and at peak in NICU. And in our study all *P* values <0.05 were considered statistically significant, and every experiment was repeated at least 3 times.

## 3 Results

### 3.1 Baseline characteristics

There are a total of 450 critically neurological patients enrolled in the study finally. In the 450 patients, 222 were had no high sodium symptoms, 228 patients were hypernatremia acquired in the NICU. And the 450 patients were divided into 3 groups according to their serum sodium levels while in the NICU: 222 had normal serum sodium (135 mmol/L < Na < 145 mmol/L), 142 had mild hypernatremia (145 mmol/L ≤ Na < 155 mmol/L), 86 had severe hypernatremia (Na ≥ 155 mmol/L). The patients with hypernatremia acquired in the NICU were 50.66% in the patients on the 1st day with normal serum sodium. Among the patients with hypernatremia acquired in the NICU, the shortest time for the patients with hypernatremia was the 1st day after entered the NICU, the longest was the 15th day after entered the ICU. And high blood sodium symptoms ranged 1 to 41 days. According to the statistics, there were 82 died, the mortality was 18.2% in the total 450 patients, and 35.96% in the patients with hypernatremia acquired in the NICU. The baseline information was listed in Table 1. And hypernatremia was significantly related with age, GCS score, serum sodium, APACHE II score, and serum creatinine (all *P* < 0.05).

**Table 1:**
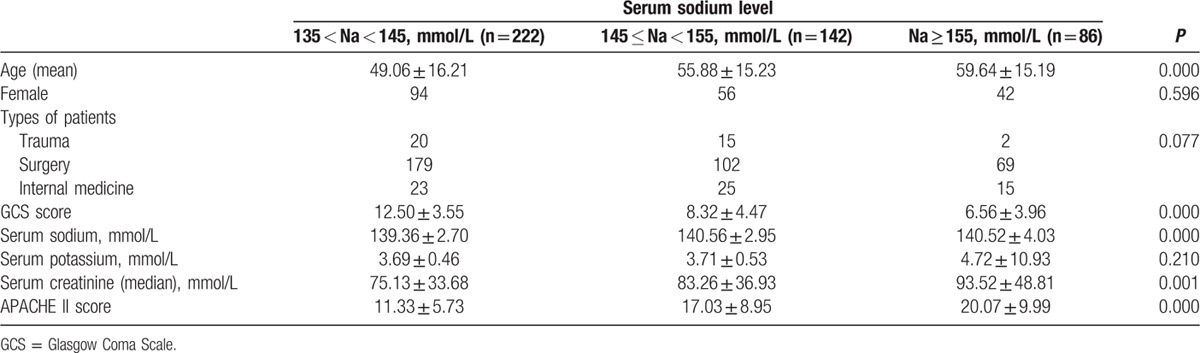
Summary of patient characteristics.

### 3.2 Treatments outcome

The treatment outcome was listed in Table 2. The results showed that the time for the mechanical ventilation and the days of stayed in ICU were both higher in the severe hypernatremia group (4.04 and 7.34 days, respectively) than other 2 groups (0.08 and 2.83, and 1.42 and 6.79, respectively). The GOS score at leaving ICU and hospital were lowest in the severe hypernatremia group (1 and 1, respectively) among the 3 groups. In addition, the mortality was highest in the severe hypernatremia group (54.95% in ICU), and the mortality rate in mild hypernatremia group was the second (10.14% in ICU). As a result, patients with severe hypernatremia levels cost more money.

**Table 2:**
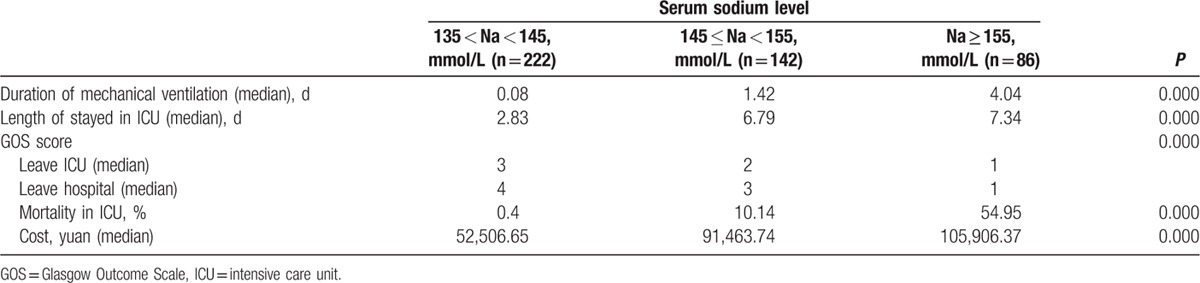
Treatments outcome of patients.

### 3.3 Associations of baseline characteristics, treatments with ICU mortality

A total of 82 patients died. The comparisons between survivors and nonsurvivors were listed in Table 3. Old ages, GCS sore, TISS score, APACHE II score, and serum sodium peak were all associated with the mortality. In the table, we could see that GCS score was significantly lower in dead patients than that in survival patients (*P* = 0.000). TISS score and APACHE II score were both higher in dead patients than that in survival patients (both *P* = 0.000). Moreover, serum sodium peak was obviously higher in dead patients than that in survival patients (*P* = 0.000).

**Table 3:**
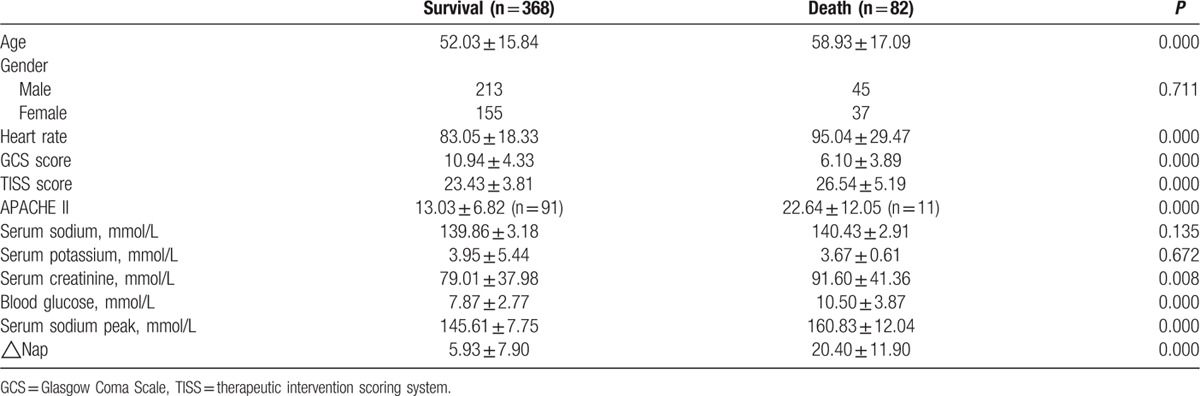
Comparison of survival and death patients.

### 3.4 The analysis of risk factors for critically neurological patients

**Figure 1:**
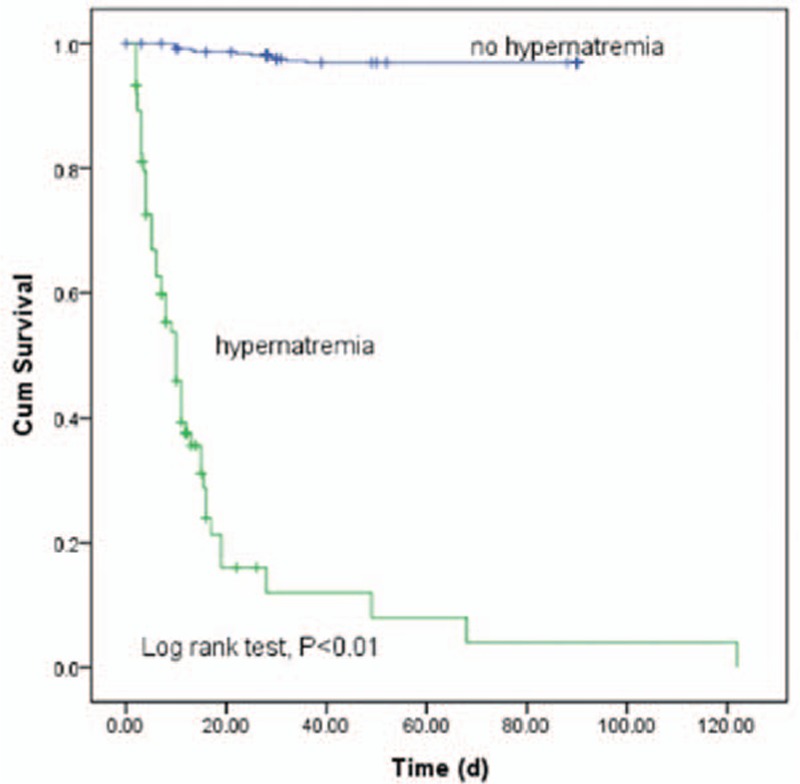
Survival curve of the patients based on the data whether had hypernatremia.

**Figure 2:**
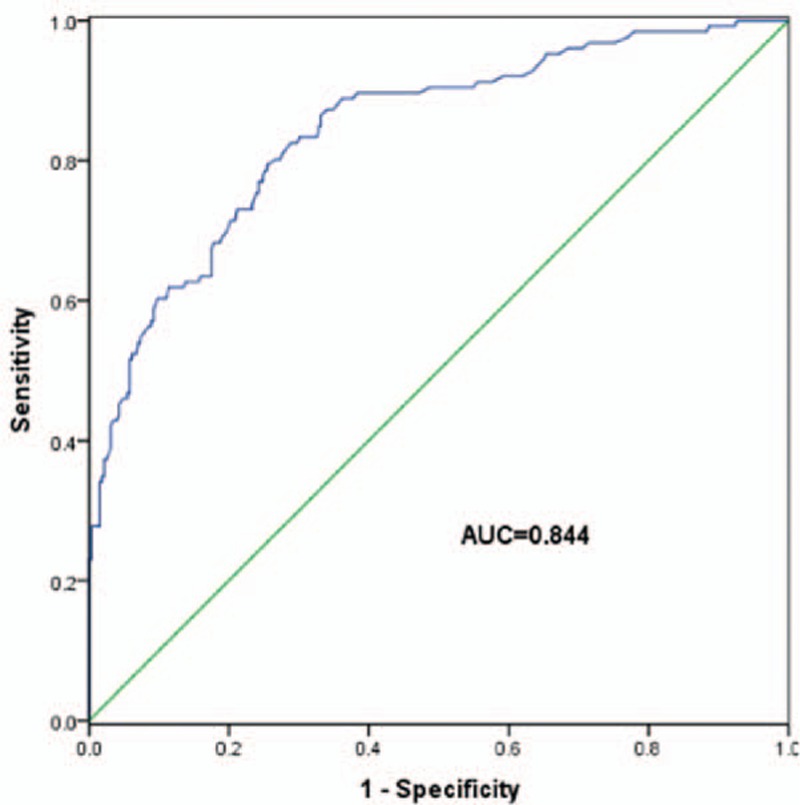
Receiver operating characteristic curve based on the correlation between peak serum sodium and the mortality of patients.

The multivariable binary logistic regression analyses were performed to analyze the risk factors of the mortality. The results were shown in Table 4. From the results, we could see that GCS score (odds ratio [OR] = 0.864, 95% confidence interval [CI] = 0.761–0.981, *P* = 0.024) and serum sodium peak (OR = 1.192, 95% CI = 1.135–1.252, *P* = 0.000) were independent factors in the mortality analysis. And higher serum sodium peak might increase the risk of mortality. Fig. 1 shows that patients with hypernatremia had worse overall survival rates than those with no hypernatremia (log-rank test, *P* < 0.05). It indicated that hypernatremia could be a predictor for the prognosis of critically neurological patients. The ROC curve (Fig. 2) between peak serum sodium and the mortality showed that the area under the curve was 0.844 with the sensitivity and specificity of 79.4% and 74.5%, respectively, and the cutoff value for the peak serum sodium was 147.55 mmol/L.

**Table 4:**
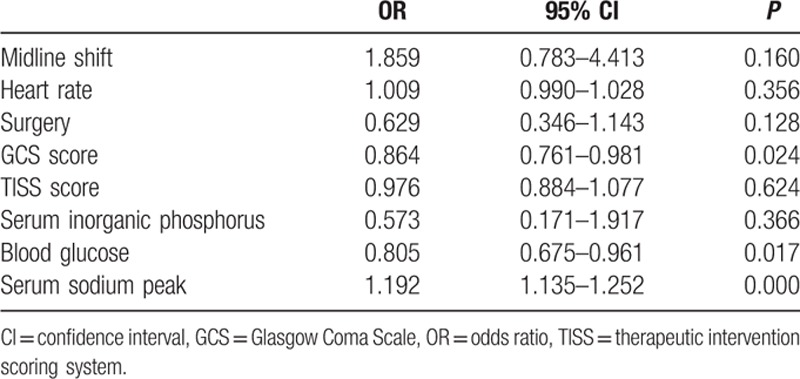
Analysis of the factors influencing the mortality.

## 4 Discussion

When serum sodium level >145 mmol/L, it could be considered as hypernatremia.^[9]^ And in the clinical research, hypernatremia is usually >150 mmol/L.^[7,10–13]^ But in our study, patients were diagnosed as hypernatremia with serum sodium level >145 mmol/L. Hypernatremia could be only developed when an increase in total body sodium or a loss of water, or both appeared.^[6]^ Moreover, osmotic diuresis because of glucose and urea can be a central causative mechanism for the development of hypernatremia among critically ill patients.^[14,15]^ Critically ill patients are often intubated, sedated, and unconscious, and their “water intake” is mainly managed by the physician, the above treatment may make them prone to the development of hypernatremia.^[16,17]^ Thus, we studied the relationship between ICU-acquired hypernatremia and the prognosis of critically neurological patients.

Hypernatremia has multiple adverse effects which may be associated with increased mortality in ICU patients.^[18]^ For example, hypernatremia can exacerbate peripheral insulin resistance,^[19]^ and hypernatremia is also correlated with various neuromuscular manifestations, such as muscle weakness and cramps.^[20]^ Neurologic impairment may be the most severe result of hypernatremia and may prolong the need for mechanical ventilation.^[9]^ Moreover, too rapid correction of chronic hypernatremia can cause cerebral edema.^[9]^ In the study of Li et al,^[21]^ 167 patients who developed a serum sodium level ≥160 mmol/L, had severe hypernatremia, and the mortality rate for the hypernatremia group was 86.8%, thus severe hypernatremia is an independent risk factor for patients with TBI. And Kolmodin et al^[22]^ found that hypernatremia in patients with TBI may be associated with increased mortality. Zhang et al^[23]^ found that NICU-acquired hypernatremia was an independent risk factor for mortality in neurologic critically ill patients. The study of Waite et al^[24]^ revealed that hypernatremia was independently associated with a 40% increase in risk of hospital mortality and a 28% increase in ICU LOS. What's more, Darmon et al^[25]^ also found that dysnatremia including mild changes in serum sodium concentration was an independent risk factor for hospital mortality. Our results were consistent with the previous studies.

In our study, hypernatremia was significantly related with age, GCS score, serum sodium, APACHE II score, and serum creatinine. And we also analyzed the relationship between ICU-acquired hypernatremia and treatment outcome. The results showed that patients with higher serum sodium level had longer mechanical ventilation time and longer length stay in ICU. Moreover, compared to patients without hypernatremia, patients with ICU-acquired hypernatremia had lower GOS score and increased ICU mortality. The results of Kaplan–Meier survival analysis showed that patients with hypernatremia had poor survival rate than those without hypernatremia. Besides old ages, GCS score, TISS score, APACHE II score, and serum sodium peak were all related with the mortality. Moreover, we also studied the prognosis value of hypernatremia in critically neurological patients. Cox regression analysis further demonstrated that the ICU-acquired hypernatremia was an independent prognostic factor for the poor survival of critically neurological patients. What's more, the results of ROC curve analysis confirmed the above findings that hypernatremia could predict survival risk of critically neurological patients.

However, there are still some limitations in our study. First, our study was a retrospective study based on the data of 1 research center. Second, our criteria could not account fully for the high incidence of hypernatremia in NICU. Finally, our patients may at a different disease state although they appeared to have a sort of dysnatremia.

In conclusion, hypernatremia is common in critically neurological patients. And we found that ICU-acquired hypernatremia is significantly associated with mortality and could be considered as an independent prognosis factor for the patients. Therefore, further correlations and ICU-specific treatment should be studied to prevent hypernatremia and increase overall survival in the future.
